# Case Report: Two cases of bronchiolar adenoma/ciliated muconodular papillary tumor characterized by significant basal cell hyperplasia and squamous metaplasia

**DOI:** 10.3389/fonc.2025.1617720

**Published:** 2025-07-25

**Authors:** Ziran Gao, Qiang Feng, Yuanyuan Wang, Ya Jiang, Dan Han, Wenmang Xu

**Affiliations:** Department of Pathology, 920th Hospital of the Joint Logistics Support Force of Chinese People’s Liberation Army, Kunming, Yunnan, China

**Keywords:** lung neoplasms, bronchiolar adenoma, ciliated muconodular papillary tumor, immunohistochemistry, basal cell hyperplasia, case report

## Abstract

**Background:**

Bronchiolar adenoma/ciliated muconodular papillary tumor (BA/CMPT) is a rare benign pulmonary tumor from the bronchiolar epithelium. Histologically, it features a continuous basal cell layer and luminal cells. Its resemblance to invasive mucinous adenocarcinoma (IMA) and acinar adenocarcinoma complicates intraoperative frozen section diagnosis. When accompanied by extensive basal cell hyperplasia (BCH) and squamous metaplasia, it may mimic sclerosing pneumocytoma or adenosquamous carcinoma. This study presents two rare BA/CMPT cases with extensive BCH and squamous metaplasia.

**Case presentation:**

Case 1: A 62-year-old female was found to have a mixed ground-glass nodule in the right lower lung lobe on CT, raising suspicion of malignancy. She received a thoracoscopic segmentectomy. Histology revealed alveolar epithelial proliferation with extensive BCH and focal squamous metaplasia. Diagnosis: distal-type BA with BCH and squamous metaplasia.

Case 2: A 67-year-old female had a solid nodule in the right lower lobe detected by CT, and a thoracoscopic wedge resection was performed. Histopathological examination revealed a lesion composed of ciliated and mucinous luminal cells overlying extensive BCH and areas of squamous metaplasia, with focal atypia observed in the basal cell layer. Diagnosis: proximal-type BA with BCH and atypical squamous metaplasia. IHC demonstrated that luminal cells in both cases expressed TTF-1, while basal cells expressed CK5/6, P63, and P40. Next-generation sequencing (NGS) did not identify any mutations or fusions in common driver oncogenes such as EGFR, BRAF, or KRAS. Postoperative follow-up showed no evidence of tumor recurrence or metastasis in either case.

**Conclusion:**

BA with extensive BCH and squamous metaplasia is rare and presents diagnostic challenges due to overlap with conditions such as sclerosing pneumocytoma, adenosquamous papilloma, adenosquamous carcinoma, mucoepidermoid carcinoma, and epithelial-myoepithelial carcinoma. Accurate diagnosis during intraoperative frozen section analysis is crucial for guiding appropriate surgical decision-making. IHC is essential for confirming the diagnosis.

## 1 Introduction

In 2002, Ishikawa (1) reported a rare pulmonary tumor originating from the bronchial epithelium and named it ciliated muconodular papillary tumor (CMPT), characterized by papillary structures and extracellular mucin deposition. In 2018, Chang et al. (2) expanded the concept by introducing bronchiolar adenoma (BA), defined as a tumor with hyperplastic bronchiolar epithelium and a continuous basal cell layer, identifying CMPT as a subtype of BA. In addition, BA is further classified into proximal-type (predominantly mucous and ciliated luminal cells, resembling bronchiolar epithelium) and distal-type (mainly type II pneumocytes or Clara cells with fewer mucous and ciliated cells, resembling terminal or respiratory bronchiolar epithelium) (2–5). Due to its histological resemblance to malignancies such as invasive mucinous adenocarcinoma (IMA) and acinar adenocarcinoma, BA faces significant challenges in diagnosis, especially during intraoperative frozen section analysis, where identifying the basal cell layer is difficult and may lead to misdiagnosis and overtreatment. This study reports two rare cases of BA with extensive BCH and squamous metaplasia, including one with atypical features. This study combines findings from the literature with clinical, histological, immunohistochemical, and molecular analyses, aiming to enhance diagnostic accuracy and guide optimal surgical decision-making.

## 2 Case description: case 1

**Figure 1:**
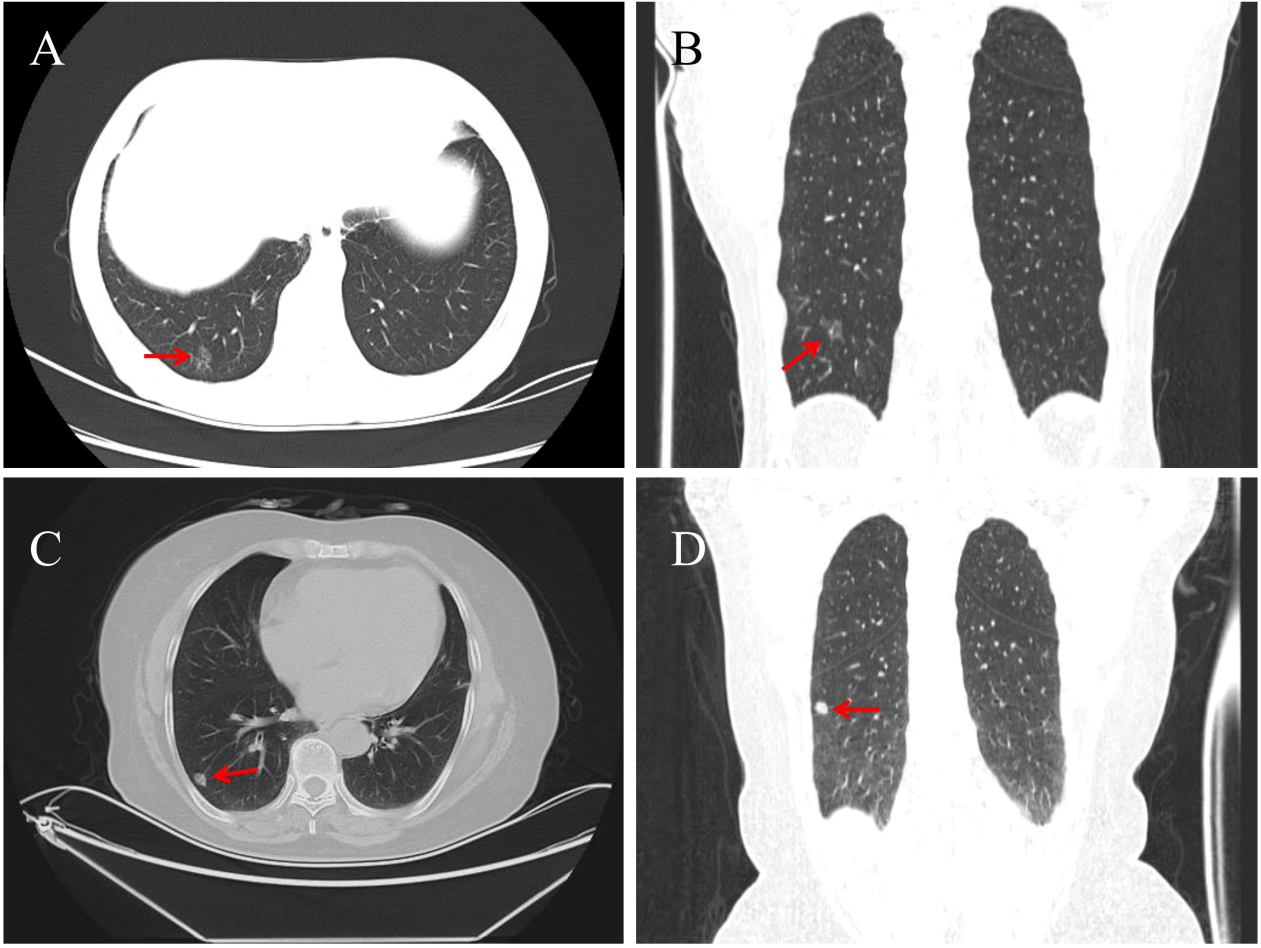
Chest CT of Case 1 shows a 14 × 11 mm pure ground-glass nodule (red arrow) in the posterior basal segment of the right lower lobe, with well-defined borders, vascular penetration, and pleural indentation [**(A)** axial; **(B)** coronal]. In Case 2, a solid 11 × 8 mm nodule (red arrow) is seen in the outer basal segment of the right lower lobe, featuring lobulation, vacuoles, and dilated draining veins [**(C)** axial; **(D)** coronal].

A 62-year-old female patient was admitted for lower back pain without symptoms of cough, sputum production, or chest tightness. She had no history of lung cancer, tuberculosis, or other major pulmonary diseases. Serum tumor markers (CEA, CYFRA21-1, SCCA, NSE, ProGRP) were within normal limits. Chest CT revealed a 14 × 11 mm mixed ground-glass nodule located in the posterior basal segment of the right lower lung lobe. The lesion exhibited well-defined borders, vascular penetration, and signs of pleural indentation, with no evidence of hilar or mediastinal lymphadenopathy (**Figures 1A, B**). Following evaluation, she received a thoracoscopic segmentectomy of the right lower lung lobe with an intraoperative frozen section diagnosis.

During surgery, an 80 mm × 45 mm × 10 mm specimen from the right lower lung lobe was examined. A gray-red, ill-defined lesion measuring 14 × 8 × 3 mm was identified approximately 10 mm from the pleura. Intraoperative frozen section analysis revealed nests and cords of uniform tumor cells growing along the alveolar stroma, with bland cytomorphology. The lesion was diagnosed as a benign lesion or a low-grade malignant. Based on these findings, mediastinal lymphadenectomy (station 9) was performed. Postoperative pathology showed no tumor metastasis.

**Figure 2:**
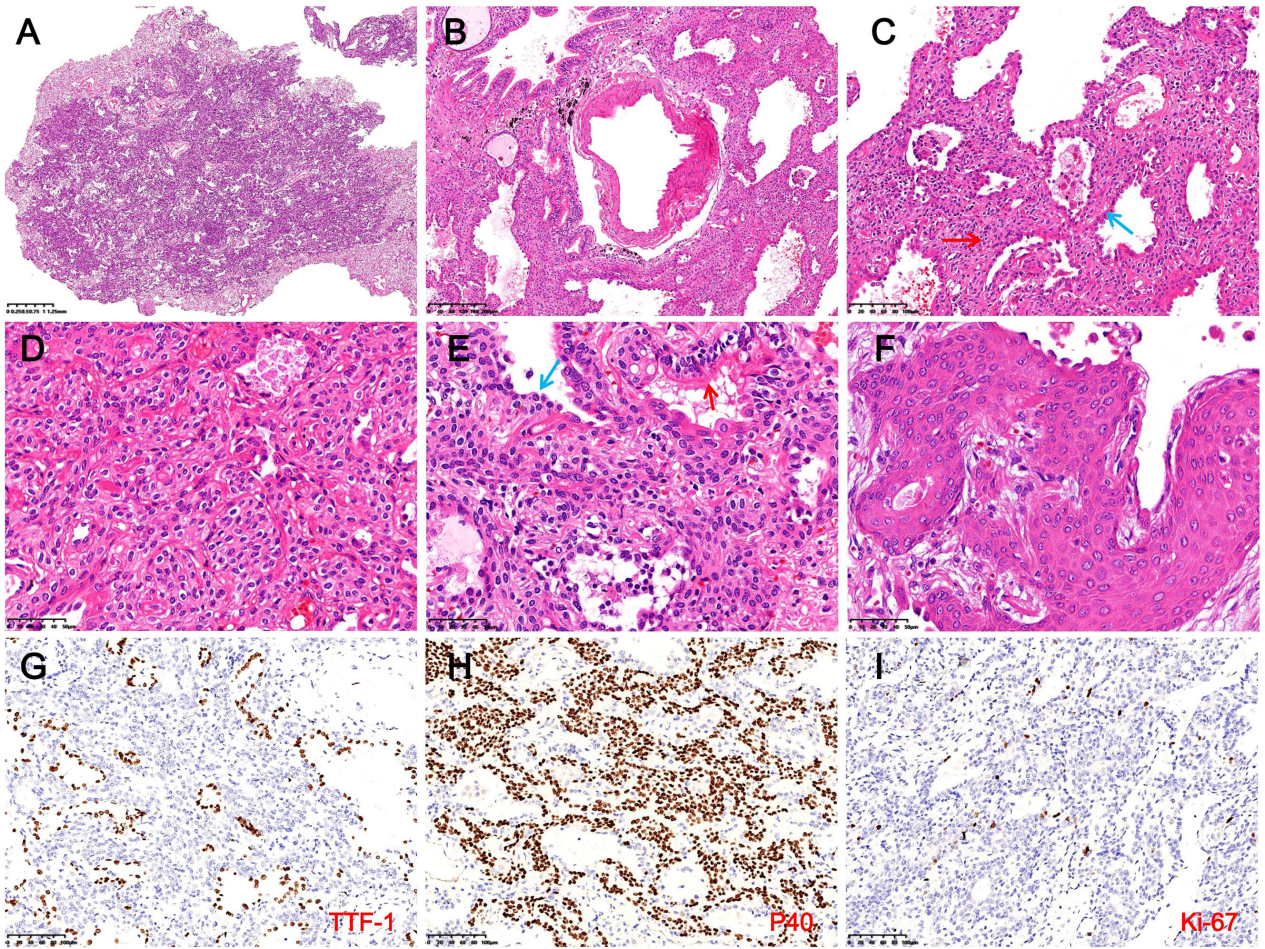
Histopathological and immunohistochemical findings in Case 1. **(A)** The tumor shows indistinct borders with surrounding lung tissue (H&E, magnification ×10). **(B)** Bronchioles and thick-walled blood vessels are observed within the tumor (H&E, magnification ×40). **(C)** In glandular and papillary regions, luminal cells (blue arrows) appear cuboidal, low columnar, or hobnail-shaped, with extensive BCH (red arrows) and foamy macrophages in alveolar spaces (H&E, magnification ×100). **(D)** In solid areas, basal cells form nests or sheets, exhibiting bland cytomorphology with eosinophilic or clear cytoplasm (H&E, magnification ×200). **(E)** In the focal area, luminal cells consist of columnar ciliated cells (red arrows) and flattened epithelial cells (blue arrows) (H&E, magnification ×200). **(F)** BCH with squamous metaplasia is evident, including visible intercellular bridges (H&E, magnification ×400). **(G)** Luminal cells express TTF-1, while hyperplastic basal cells are negative (magnification ×200). **(H)** Basal cells demonstrate diffuse expression of P40 (IHC, magnification ×200). **(I)** The Ki-67 proliferation index in hyperplastic basal cells is approximately 1% (IHC, magnification ×200).

**Figure 3:**
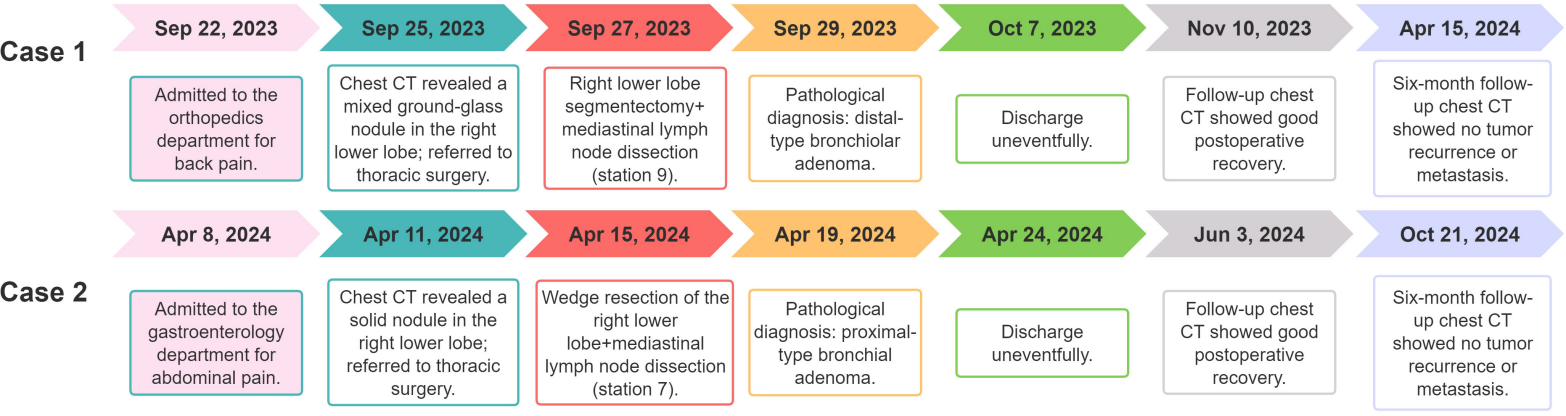
Diagnosis and treatment timeline of the cases.

Microscopy showed the tumor had ill-defined borders with surrounding lung tissue and was interspersed with bronchioles and thick-walled vessels (**Figures 2A, B**). At low magnification, glandular, papillary, and solid growth patterns were observed, with foamy macrophages in alveolar spaces. High-power views revealed adenoid and papillary areas composed of luminal and basal cells. Luminal cells were cuboidal, low columnar, or peg-shaped, with focal ciliation. Basal cells showed extensive hyperplasia with bland features, eosinophilic or clear cytoplasm, oval nuclei, fine chromatin, and absent mitoses (**Figure 2C**). Solid areas showed nests or sheets of basal cells without atypia and focal squamous metaplasia. Scattered tubules or incomplete glands lined by flattened epithelial cells were also noted (**Figures 2D–F**). Immunohistochemistry showed luminal cells positive for CK7, TTF-1, and Napsin A but negative for CK5/6, P40, and P63 (**Figure 2G**). Basal cells expressed CK5/6, P40, and P63, and were negative for TTF-1, BRAF, S-100, SMA, EMA, PR, SSTR-2, CD56, CgA, and Syn. The Ki-67 index in basal cells was ~1% (**Figures 2G, I**). DNA/RNA sequencing revealed no mutations or fusions in EGFR, ALK, ROS1, BRAF, KRAS, or other driver genes. The final diagnosis was distal-type bronchiolar adenoma with basal cell hyperplasia and squamous metaplasia. The patient recovered uneventfully, with no recurrence or metastasis after 16 months of follow-up. The clinicopathological features of the patient are summarized in **Table 1**, and the diagnostic and treatment timeline is illustrated in **Figure 3**.

**Table 1:** Clinicopathological features of two patients.

Feature	Case 1	Case 2
Age/sex	62/Female	67/Female
Location	Right lower lobe	Right lower lobe
Size (mm)	14	11
CT findings	Pure ground-glass nodules, with clear boundaries, vascular invasion and pleural retraction signs.	Solid nodules with lobulated appearance, vacuolar signs, and thickened draining veins.
Treatment	Segmentectomy + mediastinal lymphadenectomy (station 9).	Wedge resection + mediastinal lymphadenectomy (station 7).
Pathological features	Ill-defined borders; glandular, papillary, and solid patterns. Luminal cells cuboidal to low columnar with focal ciliation. BCH with bland cytology.	Well-demarcated lesion with glandular and papillary growth. Luminal cells ciliated and mucinous. Basal cells with squamous metaplasia and focal atypia.
Immunohistochemical staining	Luminal cells: CK7^+^, TTF-1^+^, Napsin A^+^; CK5/6^-^, P40^-^, P63^-^. Basal cells: CK5/6^+^, P40^+^, P63^+^; TTF-1^-^, BRAF^-^, S-100^-^, PR^-^, CgA^-^, Syn^-^. Ki-67 ≈ 1%.	Luminal cells: CK7^+^, TTF-1^+^, Napsin A^+^. Basal cells: CK5/6^+^, P40^+^, P63^+^, weak TTF-1^+^. Ki-67 ≈ 20%.
Gene alteration	None	None
Outcome	No recurrence/metastasis at 16-month follow-up.	No recurrence/metastasis at 9-month follow-up.

## 3 Case description: case 2

A 67-year-old female was admitted for abdominal pain with no symptoms of cough, sputum production, or chest tightness. She had no history of major pulmonary diseases, and serum tumor markers were normal. Chest CT showed an 11 × 8 mm solid nodule in the outer basal segment of the right lower lung lobe, showing lobulation, vacuolar signs, and dilated draining veins. No enlarged lymph nodes were observed in the hilar or mediastinal regions (**Figures 1C, D**). Following evaluation, she underwent thoracoscopic wedge resection with intraoperative frozen section analysis.

**Figure 4:**
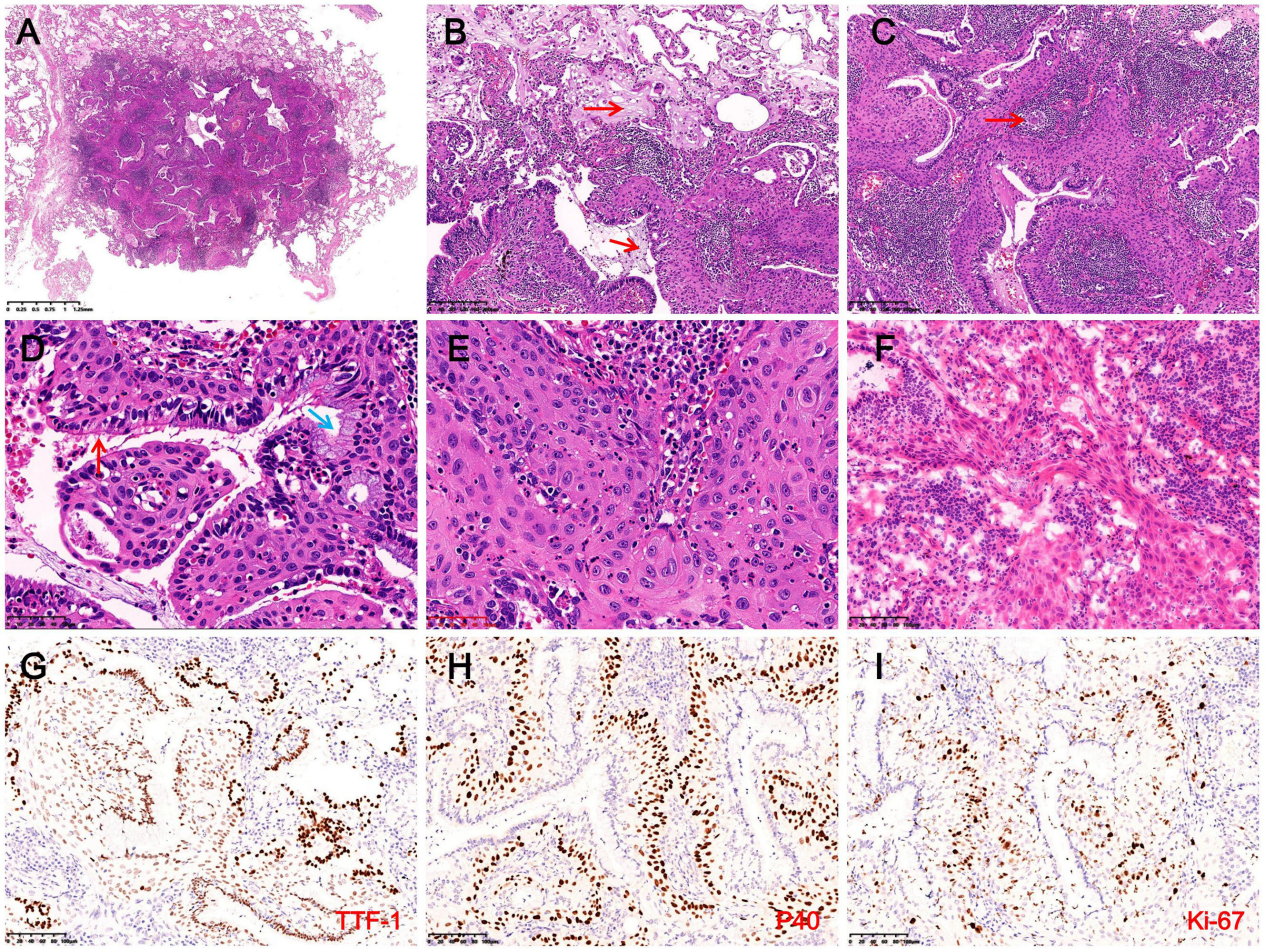
Histopathological and immunohistochemical findings in Case 2. **(A)** The tumor is well-demarcated from surrounding lung tissue and lacks a fibrous capsule (H&E, magnification ×10). **(B)** Abundant mucus (red arrows) and macrophages are observed within the tumor and surrounding alveolar spaces (H&E, magnification ×100). **(C)** Prominent stromal lymphocytic infiltration with lymphoid follicle formation (red arrows) and extensive basal cell proliferation (H&E, magnification ×100). **(D)** Luminal cells consist of ciliated cells (red arrows) and mucous cells (blue arrows), with extensively hyperplastic basal cells exhibiting squamous metaplasia arranged in a palisading pattern around glandular lumina (H&E, magnification ×400). **(E)** Focal areas exhibit disorganized basal cells with squamous metaplasia, enlarged nuclei, coarse chromatin, and cytological atypia (H&E, magnification ×400). **(F)** The intraoperative frozen section reveals basal cells forming disorganized cords, suggesting possible basement membrane invasion, with dense interstitial lymphocytic infiltration (H&E, magnification ×200). **(G)** Luminal cells strongly expressed TTF-1, while basal cells demonstrate weak expression (IHC, magnification ×200). **(H)** Basal cells exhibit diffuse expression of P40 (IHC, magnification ×200). **(I)** In hotspot areas, the Ki-67 proliferation index in basal cells reaches approximately 20% (IHC, magnification ×200).

The wedge-resected specimen measured 75 mm × 25 mm × 18 mm. A gray-white nodule (11 mm) adjacent to the pleura was noted, with no capsule and a solid, mucoid cut surface. Intraoperative frozen section analysis revealed ciliated luminal cells, mucous cells, and extensively hyperplastic basal cells with squamous metaplasia. Some areas exhibited cord-like basal cell growth with atypia and focal invasion through the basement membrane (**Figure 4F**). The intraoperative frozen section diagnosis suggested BA with BCH, squamous metaplasia, and atypical hyperplasia, with paraffin section evaluation required to exclude malignancy. Based on these findings, mediastinal lymphadenectomy (station 7) was performed, and postoperative pathological examination confirmed no evidence of metastasis.

Microscopy revealed a well-demarcated lesion with glandular and papillary growth, lacking a fibrous capsule, and traversed by thick-walled vessels. Mucin deposits and macrophages were abundant in both the tumor and surrounding alveoli, accompanied by stromal lymphocytic infiltration and the formation of lymphoid follicles (**Figures 4A–C**). The tumor exhibited a bi-layered structure of ciliated and mucous luminal cells overlying hyperplastic basal cells with squamous metaplasia. Basal cells formed palisading arrangements around glandular lumina, showing eosinophilic cytoplasm, oval nuclei, fine chromatin, and prominent nucleoli, with interspersed neutrophils (**Figure 4D**). Focal squamous metaplasia of the basal cells exhibited disorganized arrangement, irregular borders, enlarged nuclei, coarse chromatin, and occasional mitotic figures, indication cytological atypia (**Figure 4E**). IHC indicated that luminal cells were positive for CK7, TTF-1, and Napsin A, while basal cells expressed CK5/6, P40, and P63, with weak TTF-1 expression (**Figures 4G, H**). Approximately 20% of basal cells were positive for the Ki-67 proliferation index, and P53 exhibited variable nuclear expression in 20% of basal cells (**Figure 4I**). NGS revealed no mutations or fusions. The final diagnosis was proximal-type BA with BCH, squamous metaplasia, and focal atypical squamous metaplasia. The patient recovered well postoperatively, with no recurrence or metastasis at the 9-month follow-up. We recommend that both patients undergo chest CT scans at 1 month and 6 months postoperatively. If no abnormalities are detected and the condition remains stable, annual follow-up imaging is advised through the fifth postoperative year. The clinicopathological features of the patient are summarized in **Table 1**, and the diagnostic and treatment timeline is illustrated in **Figure 3**.

## 4 Discussion

BA/CMPT is a newly recognized and rare benign tumor included in the 5th edition of the WHO thoracic tumor classification (ICD-O code 8140/0) (6). It typically affects middle-aged to elderly individuals (age range: 19–84 years; mean: 68), with no sex predilection or clear association with smoking (5, 7, 8). Most cases are asymptomatic and detected incidentally during routine check-ups or unrelated evaluations (9, 10). Surgical resection is the preferred treatment for BA/CMPT, with an excellent prognosis and no reported recurrences or metastases. Since BA/CMPT typically occurs in the peripheral lung and exhibits low malignant potential, wedge resection is generally sufficient, and excessive resection and systematic lymph node sampling are unnecessary (7). This study reports two cases of incidentally detected BA/CMPT in elderly, non-smoking women who were asymptomatic and showed no abnormal serum tumor markers or lab results. Both patients underwent surgery, and follow-up revealed no recurrence or metastasis, consistent with previous reports.

BA/CMPT can occur in any lung lobe, but most often occurs in the right lower lobe, typically as a solitary, peripheral, or subpleural lesion (3, 11). Tumors range from 3.0 mm to 45 mm, mostly less than 20 mm in diameter (3, 12). CT imaging commonly shows pure or mixed ground-glass opacities or solid nodules, sometimes with cystic or cavitary features. Typical features include lobulation, vacuolar signs, vascular convergence, and spiculations, while calcifications are rare (13, 14). A slow-growing, subpleural nodule with vacuolar and vascular signs should raise suspicion for BA/CMPT (15). Proximal-type BA often appears as mucin-rich solid nodules with vacuolar signs, while distal-type BA presents as ground-glass nodules with solid components (14). However, these features are not specific to BA/CMPT and may overlap with other benign or malignant lung tumors. In this study, Case 1 shows a 14 mm distal-type BA in the posterior basal segment of the right lower lobe, appearing as a mixed ground-glass nodule with vascular penetration and pleural indentation, suggesting a high-risk lesion. Case 2 shows an 11 mm proximal-type BA in the outer basal segment of the right lower lobe, appearing as a solid nodule with lobulation, vacuolar signs, and vascular convergence. PET-CT or biopsy is advised. These cases highlight the imaging variability of BA/CMPT and the diagnostic challenge it presents.

Grossly, BA/CMPT is well-demarcated from surrounding lung tissue, lacks a distinct capsule, and exhibits a gray-white to gray-red, soft, solid cut surface, sometimes with a mucoid texture (16, 17). Histologically, it features diverse patterns—papillary, glandular, or flat—along with a characteristic bilayer structure composed of basal cells and luminal cells (ciliated, mucinous, or alveolar epithelial) (2, 6, 18). Bronchioles and thick-walled vessels are frequently observed within or near the tumor (19). The continuous basal cell layer and a bi-layered structure are critical for diagnosis. In typical cases with clear ciliated or mucinous cells, diagnosis is straightforward. However, in distal-type BA, where luminal cells are cuboidal or low columnar, IHC is often required to confirm the basal cell layer and cellular composition. Intraoperative frozen section diagnosis can be challenging due to the difficulty in identifying the bi-layered structure and ciliated cells, thus leading to potential misdiagnosis as malignant tumors. Immunohistochemical markers, such as TTF-1, Napsin A, CK5/6, p40, and Ki-67, are valuable tools for diagnosis: TTF-1 is expressed in basal and luminal cells but negative in mucinous and ciliated cells; CK5/6, p40, and p63 are strongly expressed in basal cells; and Ki-67 shows low proliferative activity (2, 19, 20).

The two cases reported here exhibit significant histological differences from classic BA/CMPT, characterized by extensive BCH and squamous metaplasia, which are rare findings. Although focal areas of BCH and squamous metaplasia have been occasionally observed in BA/CMPT, particularly in proximal-type lesions, cases exhibiting such widespread changes remain rare (9). Teng et al. (21) reported five cases of BA with BCH and squamous metaplasia, where most luminal cells were columnar with rare ciliated cells and lacked mucinous cells. The hyperplastic basal cells showed bland cytomorphology and a low Ki-67 proliferation index (<5%), resembling the features of Case 1 (distal-type BA). It is worth noting that both Case 1 and the 5 cases reported by Teng et al. (21) demonstrate negativity for TTF-1 in hyperplastic basal cells, which deviates from the typical immunophenotype of BA/CMPT. Sun et al. (7) reported a case of proximal-type BA/CMPT with squamous metaplasia exhibiting severe atypia and tumor budding, suggesting potential malignant transformation into squamous cell carcinoma. A similar pattern was observed in Case 2, which exhibited disorganized squamous cells, cytological atypia, and an elevated Ki-67 proliferation index. Wang et al. (22) found that all four proximal-type BA cases in their study displayed BCH or squamous metaplasia, including one with atypical squamous hyperplasia, whereas distal-type cases lacked these features. Additionally, Miyai et al. (23) reported a CMPT case progressing to squamous cell carcinoma. These findings suggest that proximal-type BA is more prone to BCH and squamous metaplasia compared to distal-type BA. The presence of atypical squamous changes requires thorough sampling and careful examination to rule out malignancy and avoid missed diagnosis.

In Case 1, the tumor displayed glandular, papillary, and solid nested growth patterns. The luminal cells ranged from cuboidal to low columnar in shape and were accompanied by extensive BCH characterized by bland cytomorphology, finely granular chromatin, and abundant cytoplasm. Histologically, Case 1 overlapped with sclerosing pneumocytoma, epithelial-myoepithelial carcinoma, carcinoid tumors, and primary pulmonary meningioma. Thus, differential diagnosis relied on IHC. Sclerosing pneumocytoma was excluded due to its expression of vimentin and absence of P40/P63, which contrasted with the case of BA/CMPT (24). Epithelial-myoepithelial carcinoma shares a bi-layered structure with BA/CMPT, featuring peripheral myoepithelial cells that are spindle-shaped or oval with clear or eosinophilic cytoplasm, making the two morphologically similar. However, the myoepithelial cells for epithelial-myoepithelial carcinoma express CK5/6, P63, P40, S-100, and SMA but lack TTF-1 expression, whereas hyperplastic basal cells in BA/CMPT typically express TTF-1 (25, 26). In this case, the hyperplastic basal cells were negative for TTF-1 but positive for CK5/6, P63, and P40 (myoepithelial markers), creating significant overlap in morphology and immunohistochemical results that can lead to diagnostic confusion. Carcinoid tumors were excluded due to the absence of neuroendocrine markers (CD56, CgA, Syn) in the basal cells of BA/CMPT (27). Similarly, primary pulmonary meningiomas were excluded as their tumor cells express SSTR-2, PR, and vimentin but lack P63, P40, and TTF-1 expression (28).

In Case 2, the tumor demonstrated extensive BCH, squamous metaplasia, and a papillary growth pattern, closely resembling mixed squamous cell and glandular papilloma (MSGP) in both morphology and immunophenotype. Both tumors express TTF-1, P63, and P40, making differentiation difficult, especially when squamous metaplasia is prominent. However, MSGP typically presents as a symptomatic polypoid lesion located in the proximal bronchi (29, 30), whereas BA/CMPT usually arises in the peripheral lung and is often asymptomatic, most commonly detected incidentally on imaging. In this case, the tumor was mucus-filled and composed of abundant mucinous cells and basal cells with squamous metaplasia. Focal squamous epithelial cells exhibited atypia. Extensive sampling or complete tumor excision is crucial to exclude malignant transformation into squamous cell carcinoma. Differential diagnosis includes adenosquamous carcinoma and well-differentiated mucoepidermoid carcinoma. Adenosquamous carcinoma is a bi-layered tumor composed of components from both adenocarcinoma and squamous cell carcinoma. The adenocarcinoma component may exhibit any histological subtype, while the squamous component can be either keratinizing or non-keratinizing. When composed of mucinous adenocarcinoma and non-keratinizing squamous cell carcinoma, the morphology may resemble that of Case 2. However, adenosquamous carcinoma typically shows significant cytologic atypia and stromal invasion. Well-differentiated mucoepidermoid carcinoma comprises mucinous cells, intermediate cells, and squamous cells, with a cellular composition and morphology similar to Case 2. However, it demonstrates invasive growth into surrounding tissues, the intermediate and squamous cells are negative for TTF-1, and the tumor is commonly associated with MAML2 rearrangements (31).

Multiple driver gene alterations have been identified in BA/CMPT, including mutations in BRAF, EGFR, AKT1, KRAS, and ALK rearrangements (2, 32–34). These alternations support classifying BA/CMPT as a neoplastic disease rather than a reactive or metaplastic lesion. Unlike lung adenocarcinoma, which frequently harbors EGFR mutations, the most common genetic alteration in BA/CMPT is the BRAF V600E mutation, detected in approximately 50% of reported cases (35). EGFR mutations are primarily exon 19 deletions, with occasional reports of exon 20 insertions (36). Teng et al. (21) reported BRAF V600E mutations in all five BA cases with BCH and squamous metaplasia, confirmed by BRAF immunostaining in hyperplastic basal cells, suggesting a role in tumor progression. Wang et al. (22) reported EGFR exon 19 deletions in two of four BA/CMPT cases with BCH or squamous metaplasia and BRAF V600E mutation in one case. In contrast, Sun et al. (7) reported no hotspot mutations in a BA/CMPT case with squamous metaplasia. In this study, NGS of both cases revealed no mutations or fusions in BRAF, EGFR, AKT1, or 17 other tumor-related genes. These findings suggest that the relationship between BCH/squamous metaplasia in BA/CMPT and mutations or fusions of driver genes remains unclear and requires further investigation with more case data.

In recent years, an increasing number of studies have reported malignant transformation in BA/CMPT. Chen et al. (37), Han et al. (38), Liu et al. (39), and Yang et al. (40) each reported BA case with partial or loss of the basal cell layer, showing progression to IMA. Miyai et al. (23) reported CMPT transformation into squamous cell carcinoma, while wang et al. (41) documented a BA case with features of invasive adenocarcinoma at the tumor margins, including basal cell loss, cellular atypia, and stromal invasive reactions. Zhao et al. (42) described three BA cases with peripheral glandular components lacking basal cells, demonstrating transitions from bilayered to atypical monolayered structures, along with prominent stromal fibrosis. One of these cases harbored EGFR mutations in both the preserved and atypical regions, supporting the hypothesis of malignant potential. Importantly, although previous studies have reported the potential for malignant transformation, no malignant features were observed in the present cases. Since BA was first defined in 2018, no cases of recurrence or metastasis have been reported, and the tumor is generally considered to exhibit indolent biological behavior. Sato et al. (43) proposed that CMPT is a well-differentiated pulmonary tumor with malignant potential, although it has ciliated epithelial cells. While BA/CMPT is largely considered benign, its malignant potential remains inconclusive and requires further investigation through case studies and long-term follow-up to better understand its biological behavior. Currently, no standardized follow-up duration is recommended. However, for patients who have undergone complete resection, a 5-year follow-up seems to be sufficiently safe (7).

## 5 Conclusion

BA/CMPT is a rare benign pulmonary tumor originating from the bronchiolar epithelium, characterized by a bi-layered structure consisting of proliferative bronchiolar epithelium and a continuous basal cell layer. Proximal-type BA with extensive BCH can mimic sclerosing pneumocytoma, epithelial-myoepithelial carcinoma, carcinoid tumors, and meningiomas, while distal-type BA with extensive BCH and squamous metaplasia must be differentiated from adenosquamous papilloma, adenosquamous carcinoma, and well-differentiated mucoepidermoid carcinoma. Accurate diagnosis requires integrating clinical, radiological, and immunohistochemical data. Improving the awareness of pathologists regarding the unique histological subtypes of this tumor is essential to minimize misdiagnoses during frozen section evaluations and to avoid unnecessary surgical over-resection.

## Data Availability

The original contributions presented in the study are included in the article/supplementary material. Further inquiries can be directed to the corresponding author.
